# Metabolic Effects of a 24-Week Energy-Restricted Intervention Combined with Low or High Dairy Intake in Overweight Women: An NMR-Based Metabolomics Investigation

**DOI:** 10.3390/nu8030108

**Published:** 2016-02-23

**Authors:** Hong Zheng, Janne K. Lorenzen, Arne Astrup, Lesli H. Larsen, Christian C. Yde, Morten R. Clausen, Hanne Christine Bertram

**Affiliations:** 1 Department of Food Science, Aarhus University, Kirstinebjergvej 10, Aarslev DK-5792, Denmark; 123zhenghong321@163.com (H.Z.); yde@food.au.dk (C.C.Y.); mortenr.clausen@food.au.dk (M.R.C.); 2 School of Pharmaceutical Sciences, Wenzhou Medical University, Wenzhou 325035, China; 3 Department of Nutrition, Exercise and Sports, University of Copenhagen, Frederiksberg C DK-1958, Denmark; kunchel@hotmail.com (J.K.L.); ast@nexs.ku.dk (A.A.); lehla@nexs.ku.dk (L.H.L.)

**Keywords:** ASCA, body weight, dairy, metabolome, feces, short-chain fatty acid

## Abstract

We investigated the effect of a 24-week energy-restricted intervention with low or high dairy intake (LD or HD) on the metabolic profiles of urine, blood and feces in overweight/obese women by NMR spectroscopy combined with ANOVA-simultaneous component analysis (ASCA). A significant effect of dairy intake was found on the urine metabolome. HD intake increased urinary citrate, creatinine and urea excretion, and decreased urinary excretion of trimethylamine-*N*-oxide (TMAO) and hippurate relative to the LD intake, suggesting that HD intake was associated with alterations in protein catabolism, energy metabolism and gut microbial activity. In addition, a significant time effect on the blood metabolome was attributed to a decrease in blood lipid and lipoprotein levels due to the energy restriction. For the fecal metabolome, a trend for a diet effect was found and a series of metabolites, such as acetate, butyrate, propionate, malonate, cholesterol and glycerol tended to be affected. Overall, even though these effects were not accompanied by a higher weight loss, the present metabolomics data reveal that a high dairy intake is associated with endogenous metabolic effects and effects on gut microbial activity that potentially impact body weight regulation and health. Moreover, ASCA has a great potential for exploring the effect of intervention factors and identifying altered metabolites in a multi-factorial metabolomic study.

## 1. Introduction

Obesity is a common and significant challenge to human health, not only because of a link with chronic diseases [1], but also due to a global and rapidly growing prevalence [2]. Lifestyle factors such as dietary patterns and physical activity play a key role in body weight maintenance [3,4]. Dairy products are important components in the Western dietary pattern as a good source of protein, vitamins, and minerals [5]. However, occasionally dairy consumption is proposed to have adverse effects on human health due to a high content of fat, which has been proposed as a contributor for body weight gain [6]. An evaluation based on 49 clinical trials by Lanou and Barnard [7] did not identify a conclusive association between dairy consumption and weight changes. Louie *et al.* [8] published a systematic review of 19 prospective cohort studies and reported that evidence is still not sufficient to conclude a beneficial effect of dairy intake on body weight regulation. However, based on both epidemiological and intervention studies, the review suggested a negative relationship between dairy intake and weight loss during energy restriction [9]. In addition, two meta-analyses have been published addressing the role of dairy consumption in body weight regulation. Abargouei *et al.* [10] showed that dairy intake without energy restriction may not be linked to a change in body weight, but a beneficial effect of dairy products on body weight regulation was observed in the energy-restricted trial. Chen *et al.* [11] reported that beneficial effects of dairy intake on body weight regulation and fat loss only existed in short-term or energy-restricted studies, but not in long-term or non-energy-restricted studies. Hypotheses for the potential underlying mechanisms explaining the link between intake of dairy components and body weight regulation have been proposed; for instance, whey protein is associated with a positive effect on body weight regulation due to its influence on satiety, thermogenesis, lipogenesis and body composition [12]. Dietary calcium has been reported to enhance lipolysis [13], fat oxidation [14] and fecal fat excretion [15]. Moreover, the gut microbiota stimulated by fermented dairy products [16] may also be an important environmental factor to affect body weight by the modulation of energy and lipid metabolism [17]. Recently, Nielsen *et al.* [18] reported that medium-chain fatty acids in milk can induce the expression of angiopoietin-like 4 gene (ANGPLT4), which has been reported to inhibit lipoprotein lipase and in turn regulate triglyceride metabolism [19]. To understand the link between dairy intake and body weight regulation over long-term interventions, the introduction of metabolomics approaches could contribute to elucidating the mechanisms underlying this association [20].

Therefore, in the present study, a nuclear magnetic resonance (NMR)-based metabolomics approach was applied to investigate a 24-week energy-restricted intervention with low or high intake of dairy products in overweight/obesity women, and thereby provide metabolic information for further understanding the link between dairy intake and body weight regulation. NMR spectroscopy is an attractive technique to provide the metabolite profiles of samples due to its advantages, including simple sample preparation, high reproducibility and quick analysis of urine, blood and feces.

## 2. Material and Methods

### 2.1. Subjects

A total of 38 overweight or obese women (age = 18–60 years; BMI = 28–35 kg/m^2^) were randomly assigned to a low-dairy (LD; *n* = 21) or high-dairy (HD; *n* = 17) intervention for 24 weeks with an energy-restricted diet (500 kcal energy deficit/day). The 500 kcal energy deficit was set according to the estimated energy requirement, based on body weight, sex, age and physical activity level using the Nordic Nutrition Recommendations, and with a macronutrient composition of 30 energy percentage (E%) fat, 52 E% carbohydrate and 18 E% protein. The LD diet consisted of a diet with 0–1 dairy products/day (<600 mg calcium/day) and the HD diet consisted of a diet with 4–5 dairy products/day (≈1200 mg calcium/day). The two diets were matched for relative intakes of total fat, carbohydrate, fiber and protein, and differed only in the sources due to the differences in dairy intakes. The diets were not provided by the university but based on counseling. The detailed dietary intake during energy-restricted intervention with HD or LD is provided in Table S1. At 0, 12 and 24 weeks during the intervention period, urine, blood and feces were collected and frozen for NMR-based metabolomics analysis. The present study was conducted according to the Helsinki II declaration and all procedures followed were in accordance with the Danish National Committee on Health Research Ethics. The study was registered on clinicaltrials.gov (NCT01199835). Written informed consent was obtained from the participants after they had received oral and written information about study procedures.

### 2.2. Anthropometrics and Blood Pressure

Body weight (BW) was recorded by using a digital scale accurate to 0.1 kg (Lindeltronic 8000S, Lindell’s, Malmo, Sweden). Waist and hip circumference (WC and HC) were measured with a tape measure with a precision of 0.5 cm. Ventilated-hood indirect calorimetry (Jaeger Oxycon Pro^®^, Cardinal Health Care GmbH, Hoechberg, Germany) was used to determine resting energy expenditure (REE) and respiratory quotient (RQ). Body composition (FM, fat mass; FFM, fat free mass) was measured by dual-energy X-ray absorptiometry (Lunar Prodigy DXA, Madison, WI, USA). Systolic and diastolic blood pressure (SBP and DBP) were recorded by an automatic blood pressure monitor (A & D Instruments LTD., Berkshire, UK). All measurements were repeated three times.

### 2.3. Biochemical Analysis

Fasting blood samples were collected for biochemical measurements. ABX Pentra 400 (Horiba ABX, Montpellier, France) was used to determine glucose, total cholesterol (TC), low-density lipoprotein cholesterol (LDLC), high-density lipoprotein cholesterol (HDLC), triglycerides (TG), and free fatty acids (FAA). The serum insulin level was analyzed by a solid phase and two-site chemiluminescent immunometric assay (Siemens Healthcare Diagnostics, Ballerup, Denmark) on an IMMULITE 1000 Analyzer.

### 2.4. NMR Analysis

NMR spectra were acquired using a 600.13 MHz on a Bruker Avance 600 spectrometer with a 5-mm TXI probe (Bruker BioSpin, Rheinstetten, Germany). Urine samples were thawed, vortexed and centrifuged at 10,000 g for 5 min at 4 °C, and then 500 μL of the supernatant was transferred to a 5 mm NMR tube and mixed with 100 μL of a 0.75 M phosphate buffer, which contained 0.5% sodium trimethlysilyl propionate-d_4_ (TSP) in D_2_O. The ^1^H NMR spectra were recorded at 298 K by a standard single-pulse experiment with pre-saturation of the water resonance (“zgpr”, Bruker BioSpin, Gmbh, Rheinstetten, Germany). The main acquisition parameters included, data points: 32 K, relaxation delay: 2 s, spectral width: 7288.63 Hz, and acquisition time: 2.25 s per scan. Plasma samples were also thawed, vortexed and centrifuged at 10,000 g for 10 min at 4 °C and 400 μL supernatant was transferred and mixed with 200 μL D_2_O in the NMR tube (5 mm). NMR spectra were acquired using “zgpr” pulse sequence at 310 K. In addition, to reduce the line-broadening effect of macromolecules such as lipoproteins, the “cpmg” pulse sequence, with a fixed receiver-gain value, a total spin-spin relaxation delay of 100 ms and a spin-echo delay of 1 ms, was used to better acquire signals from low-molecular weight metabolites. Lyophilized feces samples were first extracted by chloroform/methanol/water method as described in our previous study [21] into two phases, water-soluble extract (WSE) and lipid extract (LE). Then, the extract was dried and stored at −80 °C until NMR analysis. The dried WSE was dissolved in 550 µL D_2_O, 25 µL H_2_O and 25 µL D_2_O (0.05% TSP), and the dried LE was dissolved in 575 µL CDCl_3_ and 25 µL CDCl_3_ (0.05% TMS). The re-dissolved samples were vortexed thoroughly and transferred immediately into NMR tubes. NMR spectra from both WSE and LE were analyzed by using a standard “zgpr” pulse program at 298 K.

### 2.5. NMR Data Preprocessing

NMR spectra were preprocessed using auto-phase and auto-baseline corrections in the Topspin 3.0 software (Bruker BioSpin, Rheinstetten, Germany). The NMR spectra of blood were referenced to the anomeric signal of α-glucose at 5.23 ppm, while the spectra of urine and feces were referenced to TSP and TSM peaks at 0 ppm, respectively. The “icoshift” procedure was used to align NMR spectra in MATLAB (R2012a, The Mathworks Inc., Natick, MA, USA) [22]. The spectral region from 0.0 to 10.0 ppm, excluding the residual water signals from 4.7 to 5.0 ppm for urine and blood samples, the region between 0.5 and 9.5 ppm, excluding the water signals from 4.7 to 5.1 ppm for the fecal water-soluble sample, and the region between 0.5 and 8.0 ppm, excluding the chloroform signals from 7.1 to 7.3 ppm for the fecal lipid sample, were subdivided and integrated to binning data with a size of 0.01 ppm. Finally, for urine samples, the binned data were normalized to the total area of each NMR spectrum in order to minimize the dilution effect.

### 2.6. Multivariate Data Analysis

ASCA method, which combines analysis of variance (ANOVA) and simultaneous component analysis (SCA), was used in the present study. Two experimental factors, diet (LD and HD) and time (0, 12 and 24 weeks), were included. The mean spectra were used to balance the number of samples between different groups. According to ANOVA, the data matrix X (M × N, where M is the number of samples and N is the number of variables) was first split into matrices for diet and time (X_D_ and X_T_) and for interaction between the two factors (X_DT_), and a matrix with residuals (E), as described in Equation (1):

(1) $$X={\mathrm{1m}}^{T}+X_{D}+X_{T}+X_{\text{DT}}+E$$

where 1m^T^ is the overall means.

SCA then decomposes each matrix, X_D_, X_T_ and X_DT_, into a score matrix T_D_, T_T_ and T_DT_, a loading matrix P_D_, P_T_ and P_DT_, respectively, and a residual matrix E as given in Equation (2):

(2) $$X={\mathrm{1m}}^{T}+{P_{D}}^{T}T_{D}+{P_{T}}^{T}T_{T}+{P_{\text{DT}}}^{T}T_{\text{DT}}+E$$

where 1m^T^ is the overall means, P_D_ (T_D_), P_T_ (T_T_) and P_DT_ (T_DT_) are the score (loading) matrices of factor D, factor T and their interaction, respectively, and E is the residual matrix.

In this study, ASCA was performed on mean-centered and Pareto-scaled data using the ASCA toolbox [23] under MATLAB environment (R2012a, The Mathworks Inc., Natick, MA, USA) and validated using a permutation test.

### 2.7. Identification and Quantification of Metabolites

The NMR signals were carefully inspected for spectral alignment quality and excluded from models if the peaks were poorly aligned. Then, the well-aligned signals were assigned as shown in Table S2 based on our previous studies for urine and feces [21,24], and Psychogios *et al.* [25] for blood. In addition, two-dimensional (2D) ^13^C-^1^H heteronuclear single quantum coherence (HSQC) experiments were used on representative samples to confirm our tentative assignments. The relative concentrations of identified metabolites were calculated according to their peak areas by reference to the internal standard TSP or TMS concentration.

### 2.8. Statistical Analysis

Differences in baseline characteristics of the participants between LD and HD were analyzed with unpaired *t*-tests using SAS 9.2 (SAS Institute Inc, Cary, NC, USA), and *p* value < 0.05 was considered a statistically significant difference.

## 3. Results and Discussion

**Figure 1:**
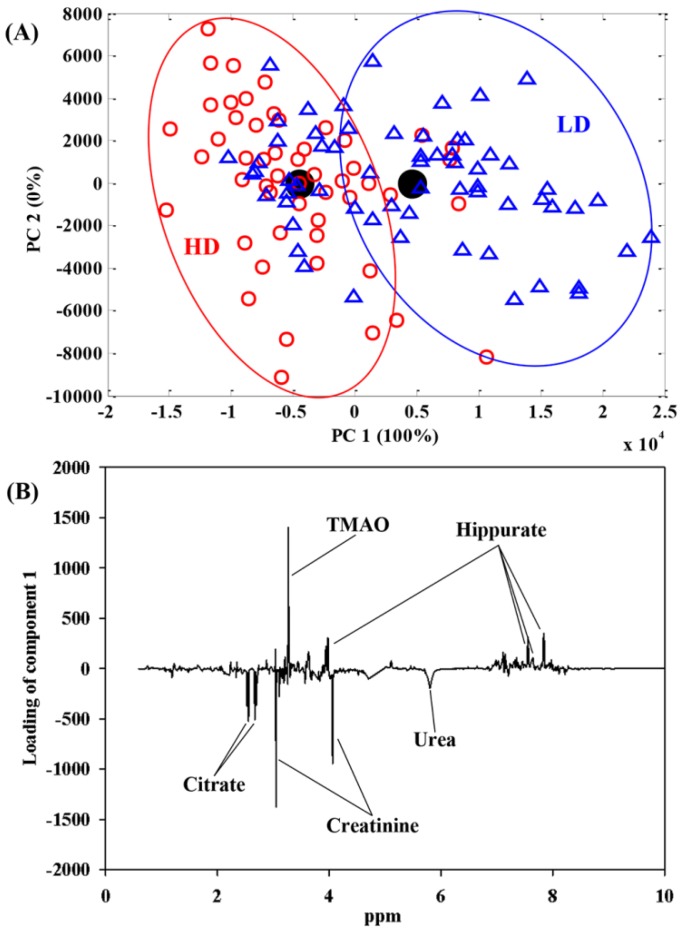
ASCA score (**A**) and loading (**B**) plots of the diet effect from NMR-based urine metabolomics in overweight/obese women who consumed the high and low dairy products. In the score plot, the scores of each sample are also included. Assignments: citrate (2.54 and 2.68 ppm); creatinine (3.05 and 4.06 ppm); TMAO (3.27 ppm); hippurate (3.97, 7.55, 7.64 and 7.84 ppm); urea (5.80 ppm).

**Figure 2:**
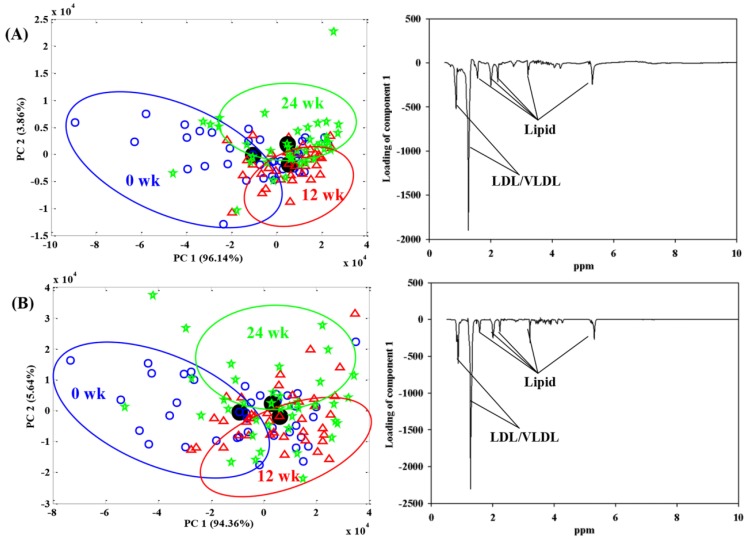
ASCA score and loading plots from NMR-based blood metabolomics in overweight/obese women who consumed the high and low dairy products at 0, 12 and 24 weeks: (**A**) “zgpr” data; (**B**) “cpmg” data. In the score plot, the scores of each sample are also included. Assignments: LDL/VLDL (0.85 and 1.27 ppm); lipid (1.57, 2.01, 2.23, 3.21 and 5.30 ppm).

In recent years, NMR-based metabolomics has been used as a potential tool not only to identify dietary biomarkers [26,27,28] but also to explore the metabolic effects of dietary interventions [29,30,31]. This study was conducted to investigate the changes in the human metabolome of urine, blood and feces samples after intake of an energy-restricted LD or HD diet for 24 weeks. The identification of the exact metabolic alterations associated with dairy products intake under energy restriction have large implications because it would facilitate our understanding of the mechanisms by which dairy products may assist in body weight regulation. There was no significant difference between treatment groups at baseline (Table 1). Metabolomics data were analyzed using ASCA, which enables the inclusion of underlying factors such as diet, time and their interaction, and facilitates the interpretation of the variation induced by different factors as compared with a conventional PCA model [32]. Furthermore, through orthogonal projection of the samples onto the scores of the time and diet effects, we were able to assess the magnitude of these effects [33]. Table 2 lists statistically significant values obtained from 10,000 permutation tests in ASCA models, including the effects of diet, time and their interaction (diet x time). The corresponding ASCA score and loading plots derived from significant models are illustrated in Figure 1 and Figure 2 for the diet and time effects on the urinary (*p* = 0.004) and blood (*p* = 0.003 for “zgpr” data; *p* = 0.0005 for “cpmg” data) metabolome, respectively, as well as Figure S1 for the diet effects on the fecal metabolome, which tended to be significant (*p* = 0.09 for WSE; *p* = 0.06 for LE). The ASCA model results revealed a significant diet effect on the urinary metabolome, but not on the blood metabolome (Table 2). These results are in agreement with a previous study [24], where we found that the diet effect is more evident in the urinary metabolome than in the blood metabolome. In addition, Walsh *et al.* [34] likewise showed that urine samples are more sensitive in reflecting dietary effects than plasma or saliva.

Figure 1 shows that the diet effect on the urinary metabolome can be ascribed to an increase in the urinary excretion of citrate, creatinine and urea, and a decrease in hippurate and TMAO after intake of the HD diet relative to the LD diet. Urinary citrate might originate directly from citrate-containing foods, such as cranberry juice [35], tea [36], and grape juice [25]. Citric acid is also a main organic acid in dairy products by Tormo and Izco [37]. Thus, an increase in urinary citrate level in the HD group may originate directly from the HD intake. However, citrate is also endogenously synthesized as a major tricarboxylic acid (TCA) cycle intermediate, and the higher urinary excretion of citrate associated with HD consumption could also reflect that HD enhances the mitochondrial TCA cycle. Such an endogenous metabolic effect is in agreement with a recent study on mice, which revealed that whey protein diets increased the urinary excretion of TCA cycle intermediates including citrate and thereby provided less substrate for lipogenesis, which resulted in decreased lipid accretion and body weight [38]. Urea, a major product of protein catabolism, is synthesized from the urea cycle in liver and excreted in the urine. Urinary urea has been validated as a biomarker of protein ingestion [39]. An increased urinary urea was observed after the HD intake but not after LD diet even though the two diets were similar in protein content. In a study comparing whey, casein and skimmed milk intake, it was also observed that protein source affected urinary urea excretion differently as whey intake resulted in lower urea excretion [40], and also other studies have reported that protein source affects urinary urea excretion [41]. In addition, HD intake resulted in a higher urinary creatinine level relative to the LD intake. Together, these findings suggest that the HD diet regulates protein catabolism differently from the LD diet, which may help maintain lean body mass better than the LD diet under dietary energy restriction, since urinary creatinine level has been validated as a marker of lean body mass [42]. Zemel *et al.* [13] also reported that a high dairy diet reduced lean body mass loss (*p* < 0.001) compared with the low dairy diet during energy restriction. Urea and creatinine are two main markers of kidney function [43]. The impact of differences in urea and creatinine excretion on renal function remains unknown. The metabolite TMAO originates from choline metabolism in the gut flora. Choline-containing diets such as milk, egg, meat and fish can increase the production and excretion of TMAO. It has been proposed that TMAO raises the risk of cardiovascular disease (CVD) [44], but the link is a matter of dispute [45,46]. Intriguingly, a reduction in the urinary TMAO excretion after the HD consumption was observed in the present study, which indicates that the HD intake may regulate the gut flora to decrease TMAO production. In a metabolomics study comparing cheese and milk intake, we also identified an effect of dairy intake on urinary TMAO excretion [21]. In the present study, a lower urinary hippurate was observed in the HD group than the LD group. Hippurate is synthesized as a glycine conjugate of benzoic acid, which involves microbial metabolism and finally excreted into urine. Urinary hippurate has been reported as a marker of the gut microbial activity, but variations in hippurate excretion may also be attributed to alterations in glycine or CoA availability [47]. In addition, urinary hippurate level is also influenced by diet, gender, age, and disease [47]. In the present study, the lower urinary hippurate excretion observed in the HD group likely reflects an effect on the gut microbiota’s activity in response to dairy intake.

For the blood metabolome there was a significant effect of the energy restriction (time effect), which could be ascribed to changes in the lipid and lipoprotein profile as shown in the ASCA loading plots in Figure 2. These findings corroborate a recent metabolomics study conducted during a 6-week calorie restriction [48], and propose beneficial effects of weight loss on CVD risk. However, an effect of dairy intake on the blood metabolome was not detectable in the current study. A possible explanation for this result is that the energy restriction had a substantially greater impact on the blood metabolome, masking any potential effect of the dairy intake on the blood metabolome.

In addition, the ASCA results indicated that dairy intake affected microbial-related metabolites in the fecal metabolome, including SCFAs (acetate, butyrate and propionate) and malonate (Figure S1). However, the diet effect of this ASCA model was not significant (Table 2), which may be attributed to the energy restriction in the study, as we found that the levels of fecal SCFAs and malonate were significantly altered after milk or cheese consumption in a previous study without energy restriction [21]. St-Onge *et al.* [16] also reported that fermented dairy products can increase the amount of gut microbiota and in turn produce more SCFAs. Moreover, dairy intake also affected cholesterol, glycerol and lipid levels in feces as shown in Figure S1, which may also be regulated by gut microbiota-derived SCFAs. For instance, acetate is a precursor of cholesterol synthesis, whereas propionate and butyrate may inhibit cholesterol formation and reduce fat absorption [49,50]. Another plausible explanation for the higher fecal fat excretion with the HD diet could be that dairy calcium binds fat, which is then excreted in the feces [15]. Thus, lipid and microbial metabolism appear to be affected by dairy intake.

## 4. Conclusions

In the present study, using ASCA to separate diet and time effects and thereby reduce complexity of metabolomics data, we explored the effect of dairy intake on the human metabolome after a long-term energy restriction intervention. The ASCA model showed a significant diet effect on the urinary metabolome, a significant time effect on the blood metabolome and a borderline diet effect on the fecal metabolome. We found that the energy restriction affected blood lipid profile, implying beneficial effects on CVD risk. The dairy-induced changes reflected in the metabolome involved protein catabolism, endogenous energy, lipid and gut microbial metabolism, which likely impact maintenance of lean body mass, fat deposition and gut health. Consequently, the present study, which has high translational value because of the long duration of the intervention period, has provided new insight into metabolic effects associated with dairy intake. However, further studies are needed to corroborate the detailed relationship between the specific metabolic effects identified, body weight regulation and human health.

## Figures and Tables

**Table 1:** Characteristics of the participants at baseline during energy-restricted intervention with high or low dairy products ^a^.

	LD (*n* = 21)	HD (*n* = 17)	*p*
Age (year)	45.2 ± 2.9	41.3 ± 2.7	0.13
BMI (kg/m^2^)	30.4 ± 1.5	30.0 ± 1.6	0.68
FM (kg)	36.1 ± 2.2	36.3 ± 2.6	0.91
FFM (kg)	44.0 ± 2.3	47.2 ± 2.3	0.50
WC (cm)	96.3 ± 3.0	97.5 ± 3.0	0.66
HC (cm)	110.9 ± 2.5	109.5 ± 2.7	0.54
REE (kcal)	1389.0 ± 14.3	1447.8 ± 12.9	0.34
RQ	0.83 ± 0.17	0.83 ± 0.21	0.43
SBP (mmHg)	120.2 ± 3.7	119.1 ± 3.6	0.81
DBP (mmHg)	80.0 ± 3.3	79.5 ± 3.0	0.89
TC (mmol/L)	5.2 ± 1.0	5.2 ± 0.8	0.81
LDLC (mmol/L)	3.1 ± 0.9	3.1 ± 0.8	0.87
HDLC (mmol/L)	1.3 ± 0.4	1.4 ± 0.6	0.64
TG (mmol/L)	1.3 ± 0.8	1.2 ± 0.8	0.75
Plasma glucose (mmol/L)	5.6 ± 0.8	5.5 ± 0.6	0.73
Serum insulin (pmol/L)	52.7 ± 4.9	42.6 ± 5.8	0.28
FFA (μmol/L)	726.9 ± 15.9	619.4 ± 14.7	0.18

^a^ Data are presented as mean ± SEM. Difference analysis was made by unpaired T-tests. LD, low dairy intake; HD, high dairy intake; BMI, body mass index; FM, fat mass; FFM, fat free mass; WC, waist circumference; HC, hip circumference; REE, resting energy expenditure; RQ, respiratory quotient; SBP, systolic blood pressure; DBP, diastolic blood pressure; TC, total cholesterol; LDLC, low-density lipoprotein cholesterol; HDLC, high-density lipoprotein cholesterol; TG, triglycerides; FFA, free fatty acids.

**Table 2:** *p* values of ASCA models by 10,000 permutations test.

Samples	Diet	Time	Diet × Time
Urine	0.004	0.66	0.25
Blood ZGPR ^a^	0.39	0.003	0.96
Blood CPMG ^b^	0.89	0.0005	0.95
Feces WSE ^c^	0.09	0.52	0.98
Feces LE ^d^	0.06	0.33	0.87

^a^ NMR data were obtained using a “zgpr” pulse sequence; ^b^ NMR data were obtained using a “cpmg” pulse sequence; ^c^ water-soluble extract; ^d^ lipid extract.

## Supplementary Materials

The following are available online at http://www.mdpi.com/2072-6643/8/3/108/s1, Figure S1: ASCA score and loading plots from NMR-based fecal metabolomics in overweight/obesity women consumed the high (**○**) and low (**✰**) dairy products: (A) score plots based on chloroform soluble extraction; (B) score plots based on water soluble extraction. Assignments: acetate (1.92 ppm); propionate (1.05 and 2.16 ppm); butyrate (0.90, 1.56 and 2.20 ppm); malonate (3.11 ppm); glycerol (3.56, 3.65 and 3.77 ppm); cholesterol (0.91 and 1.04 ppm); lipid (0.80, 1.22, 1.55 and 2.27 ppm), Table S1: Dietary intake during energy-restricted intervention with high or low dairy products, Table S2: Assignment of metabolites in 1H NMR spectra of urine, blood and feces in overweight and obesity women.
