# Supplementary material for: Metabolic Effects of a 24-Week Energy-Restricted Intervention Combined with Low or High Dairy Intake in Overweight Women: An NMR-Based Metabolomics Investigation
Source: Nutrients. 2016 Feb 23;8(3):108. doi: 10.3390/nu8030108 (PMC4808838; doi:10.3390/nu8030108)
Supplement: Supplementary file 1 [file nutrients-08-00108-s001.docx]

Supplementary Materials: Metabolic Effects of a 24-Week Energy-Restricted Intervention Combined with Low or High Dairy Intake in Overweight Women: An NMR-Based Metabolomics Investigation

Hong Zheng, Janne K. Lorenzen, Arne Astrup, Lesli H. Larsen, Christian C. Yde,
Morten R. Clausen and Hanne Christine Bertram


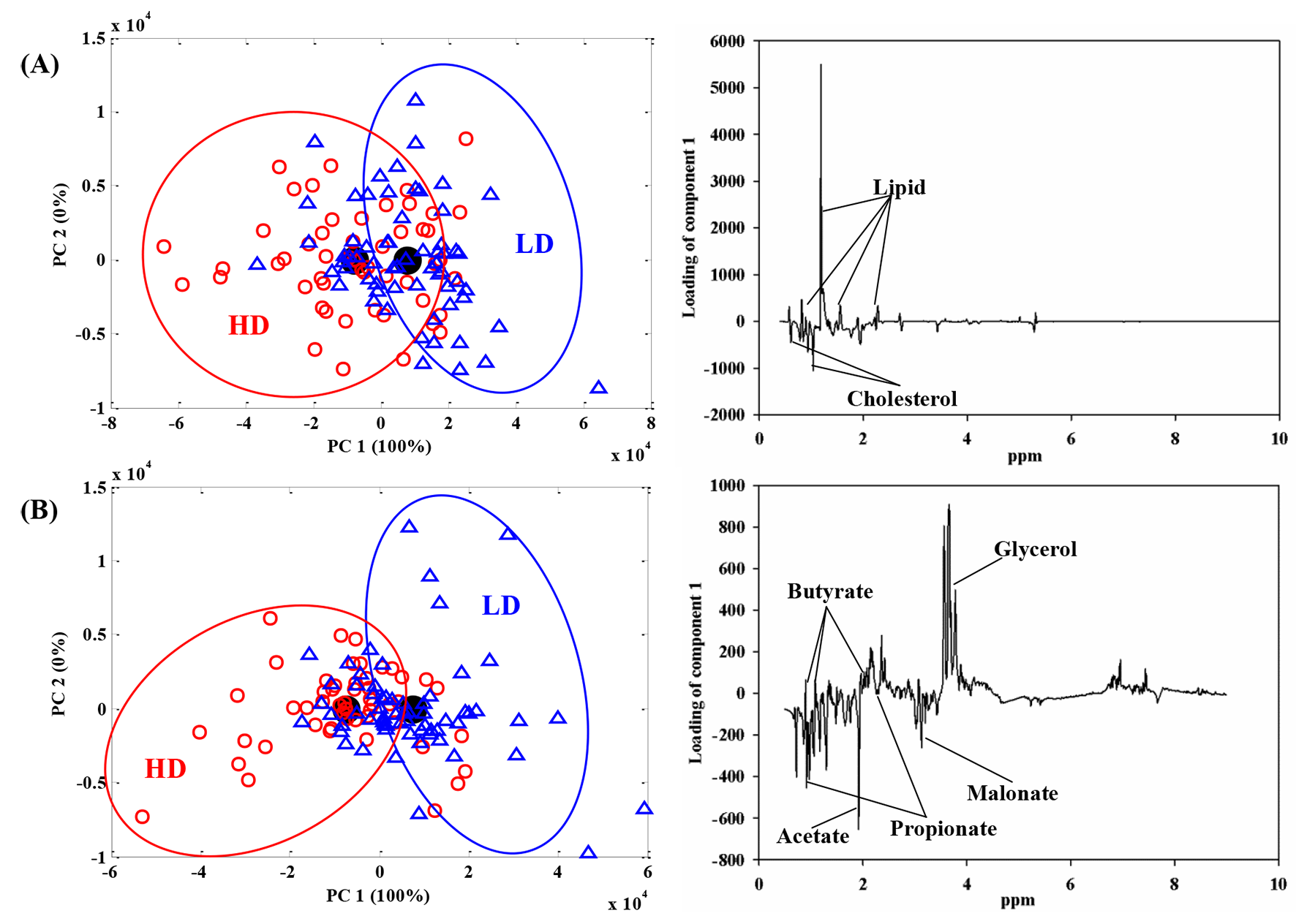


**Figure S1.** ASCA score and loading plots from NMR-based fecal metabolomics in overweight/obesity women consumed the high (**○**) and low (**△**) dairy products: (**A**) score plots based on chloroform soluble extraction; (**B**) score plots based on water soluble extraction. Assignments: acetate (1.92 ppm); propionate (1.05 and 2.16 ppm); butyrate (0.90, 1.56 and 2.20 ppm); malonate (3.11 ppm); glycerol (3.56, 3.65 and 3.77 ppm); cholesterol (0.91 and 1.04 ppm); lipid (0.80, 1.22, 1.55 and 2.27 ppm).

**Table S1.** Dietary intake during energy-restricted intervention with high or low dairy products ^1^.

|  | **HD^a^** | | | | **LD^b^** | | | | **P0^c^** | **P12^d^** | **P24^e^** |
| --- | --- | --- | --- | --- | --- | --- | --- | --- | --- | --- | --- |
|  | **0 week** | **12 weeks** | **24 weeks** | **P** | **0 week** | **12 weeks** | **24 weeks** | **P** |  |  |  |
| Energy intake (MJ/day) | 8663 ± 46a | 6644 ± 35b | 6363 ± 31b | 0.0003 | 8907 ± 47a | 6499 ± 40b | 6165 ± 33b | <0.0001 | -^f^ | - | - |
| Calcium (mg/day) | 993 ± 20b | 1341 ± 16Aa | 1333 ± 16Aa | 0.004 | 953 ± 15a | 579 ± 15Bb | 545 ± 14Bb | <0.0001 | - | <0.0001 | <0.0001 |
| Protein (E%) | 18 ± 2b | 22 ± 1a | 21 ± 2a | 0.0008 | 17 ± 2b | 20 ± 2a | 20 ± 2a | 0.003 | - | - | - |
| Carbohydrate (E%) | 47 ± 2 | 45 ± 2 | 48 ± 2 | - | 47 ± 2 | 45 ± 2 | 46 ± 2 | - | - | - | - |
| Fat (E%) | 34 ± 2 | 32 ± 2 | 30 ± 2 | - | 33 ± 2 | 32 ± 2 | 32 ± 2 | - | - | - | - |
| Fiber (g/day) | 24 ± 2 | 19 ± 2 | 21 ± 2 | - | 23 ± 3 | 23 ± 4 | 21 ± 2 | - | - | - | - |
| SFA (%) | 28 ± 3 | 23 ± 3A | 22 ± 3A | - | 28 ± 3a | 16 ± 3Bb | 16 ± 2Bb | <0.0001 | - | 0.002 | 0.03 |
| MUFA (%) | 26 ± 3a | 19 ± 2b | 18 ± 3b | 0.008 | 26 ± 3a | 19 ± 2b | 19 ± 3b | 0.005 | - | - | - |
| PUFA (%) | 12 ± 2a | 8 ± 1Bb | 8 ± 2Bb | 0.0001 | 12 ± 2 | 11 ± 2A | 10 ± 1A | - | - | 0.002 | 0.05 |

^1^ Data are presented as mean ± SE. ^a^ high dairy; ^b^ low dairy; ^c^ difference between HD and LD at 0 week; ^d^ difference between HD and LD at 12 weeks; ^e^ difference between HD and LD at 24 weeks; ^f^ no significant difference. Statistical comparisons were performed by unpaired t-tests. E%, energy percentage; SFA, saturated fatty acid; MUFA, monounsaturated fatty acid; PUFA, polyunsaturated fatty acid. Different lowercase and uppercase letters represent statistically significant differences among different times in the same diet group and between two diets at the same period, respectively.

**Table S2.** Assignment of metabolites in ^1^H NMR spectra of urine, blood and feces in overweight and obesity women.

| **No.** | **Metabolites** | **Integrated Region (ppm)** | **δ ^1^H (Multiplicity) ^a^** | **Moieties** |
| --- | --- | --- | --- | --- |
| **Urine** | | | | |
| 1 | Citrate | 2.53–2.56 | 2.54 (d); 2.68 (d) | half-CH_2_; half-CH_2_ |
| 2 | Creatine | 3.91–3.93 | 3.04 (s); 3.92 (s) | CH_3_; CH_2_ |
| 3 | Creatinine | 4.05–4.07 | 3.05 (s); 4.06 (s) | CH_3_; CH_2_ |
| 4 | TMAO | 3.26–3.28 | 3.27 (s) | N(CH_3_)_3_ |
| 5 | Hippurate | 7.54–7.57 | 3.97 (d); 7.55 (t); 7.64 (t); 7.84 (d) | CH2; CH3/CH5; CH4; CH2/6 |
| 6 | Urea | 5.70–5.90 | 5.80 (s, broad) | NH_2_ |
| **Blood** | | | | |
| 1 | LDL/VLDL ^c^ | 0.80–0.90 | 0.85(m); 1.27(m) | CH_3_; CH_2_ |
| 2 | Blood lipid ^b^ | 5.27–5.33 | 1.57 (m); 2.23 (m); 3.21 (m); 5.30(m) | CH_2_; CH_2_; CH_2_; =CH |
| **Feces water-soluble extract** | | | | |
| 1 | Propionate | 1.04–1.07 | 1.05 (t); 2.16 (q) | CH_3_; CH_2_ |
| 2 | Butyrate | 1.53–1.59 | 0.90 (t); 1.56 (tq); 2.20 (t) | CH_3_; CH_2_; CH_2_ |
| 3 | Acetate | 1.91–1.93 | 1.92 (s) | CH_3_ |
| 4 | Malonate | 3.10–3.12 | 3.11 (s) | CH_2_ |
| 5 | Glycerol | 3.64–3.67 | 3.56 (m); 3.65 (m); 3.77 (tt) | (CH_2_)_2_; (CH_2_)_2_; CH |
| **Feces lipid extract** | | | | |
| 1 | Lipid | 1.17–1.30 | 0.80 (m); 1.22 (m); 1.55 (m); 1.95 (m); 2.27 (m); 5.27 (m) | CH_3_; CH_2_; CH_2_; CH_2_; CH_2_; =CH |
| 2 | Cholesterol | 1.03–1.05 | 0.91 (m); 1.04 (m) | - |

^a^ s, singlet; d, doublet; t, triplet; q, quartet; tt, triplet of triplets; tq, triplet of quartets; m, multiplet; ^b^ integrated from “cpmg”; ^c^ integrated from “zgpr”.
